# Dissecting the microenvironment around biosynthetic scaffolds in murine skin wound healing

**DOI:** 10.1126/sciadv.abf0787

**Published:** 2021-05-26

**Authors:** Chen Hu, Chenyu Chu, Li Liu, Chenbing Wang, Shue Jin, Renli Yang, Shengan Rung, Jidong Li, Yili Qu, Yi Man

**Affiliations:** 1 Department of Oral Implantology and State Key Laboratory of Oral Diseases and National Clinical Research Center for Oral Diseases, West China Hospital of Stomatology, Sichuan University, Chengdu, Sichuan 610041, China.; 2 State Key Laboratory of Biotherapy, West China Hospital, Sichuan University, Collaborative Innovation Center for Biotherapy, Chengdu, Sichuan 610041, China.; 3 Research Center for Nano-Biomaterials, Analytical, and Testing Center, Sichuan University, Chengdu 610064, China.

## Abstract

The structural properties of biomaterials play crucial roles in guiding cell behavior and influencing immune responses against the material. We fabricated electrospun membranes with three types of surface topography (random, aligned, and latticed), introduced them to dorsal skin excisional wounds in mice and rats, and evaluated their effects on wound healing and immunomodulatory properties. An overview of different immune cells in the microenvironment with the help of single-cell RNA sequencing revealed diverse cellular heterogeneity in vivo. The time course of immune response was advanced toward an adaptive immunity–dominant stage by the aligned scaffold. In mice without mature T lymphocytes, lack of wound-induced hair neogenesis indicated a regulatory role of T cells on hair follicle regeneration. The microenvironment around scaffolds involved an intricate interplay of immune and cutaneous cells.

## Teaser

Scaffolds with aligned fibers promoted a T cell-dominant adaptive immune response in murine skin wound healing.

## INTRODUCTION

Biomaterials and devices implanted in the body have a broad spectrum of clinical applications like tissue regeneration and cell transplantation (*1*, *2*). Among the components of a cellular microenvironment, structural features (macroscale, microscale, and nanoscale features) play critical roles in guiding cell behavior (*3*). Electrospinning technology has been widely used in preparing scaffolds owing to its simplicity, the capacity to form fibers on a micro- and nanoscale, structural control of the electrospun membranes, and cost-effectiveness (*4*). Electrospun nanofibers can be assembled into well-ordered nanofiber meshes with different morphologies, e.g., in a parallel alignment or latticed patterns of nanofibers (*5*, *6*), and have been used for the regeneration of diverse tissues such as skin and bone (*6*, *7*). Upon implantation of a material, cells of both the innate and adaptive immune systems play a role in the host response (*8*, *9*). Previous studies have illustrated type 1 (proinflammatory) immune polarization driven by T helper 1 (T_H_1) cells, and the induced proinflammatory M1 activation of macrophages [stimulated by interleukin-2 (IL-2) and interferon-γ (IFN-γ) from T_H_1 cells]. Conversely, in type 2 immune response, T_H_2 cells produce cytokines, such as IL-4 and IL-13, which regulate the polarization of macrophages toward an anti-inflammatory M2 activation (*10*, *11*). More recently, a type 17 immune response was reported to promote chronic fibrosis in the tissue around the implants (*12*, *13*). In summary, previous studies had often focused on certain types of immune cells, such as macrophages, and explored their roles in host response. However, immune response is jointly regulated by various immune cells, whose phenotypes and functions are dictated by external and internal signals. An overview of different immune cells in the microenvironment will aid in the comprehensive understanding of the immune responses elicited by scaffolds. Technological advances, such as single-cell RNA sequencing (scRNA-seq) (*14*, *15*), have enabled cell population, cell function, and the nuances of their phenotypes in vivo to be studied at a high resolution. By changing the collector, we developed poly(lactic-*co*-glycolic acid)–fish collagen (PLGA-FC) hybrid electrospun scaffolds with three types of surface topography, i.e., groups with randomly oriented fibers (random group), mesh-like topography and randomly oriented fibers in microscale (latticed group), and aligned fibers (aligned group). We explored the regenerative outcomes of these scaffolds in rat/mouse dorsal skin excisional wounds and evaluated their immunomodulatory properties. The microenvironment around the scaffolds and Ctrl was investigated using scRNA-seq. Heterogeneity of keratinocytes, fibroblasts, and immune cell populations, cellular functions, and their interactions were explored in vivo. As far as we were concerned, this is the first study deciphering the overall immune microenvironment around cutaneous scaffold using scRNA-seq.

## RESULTS

### Evaluation of wound healing in a rat skin wound model

**Fig. 1:**
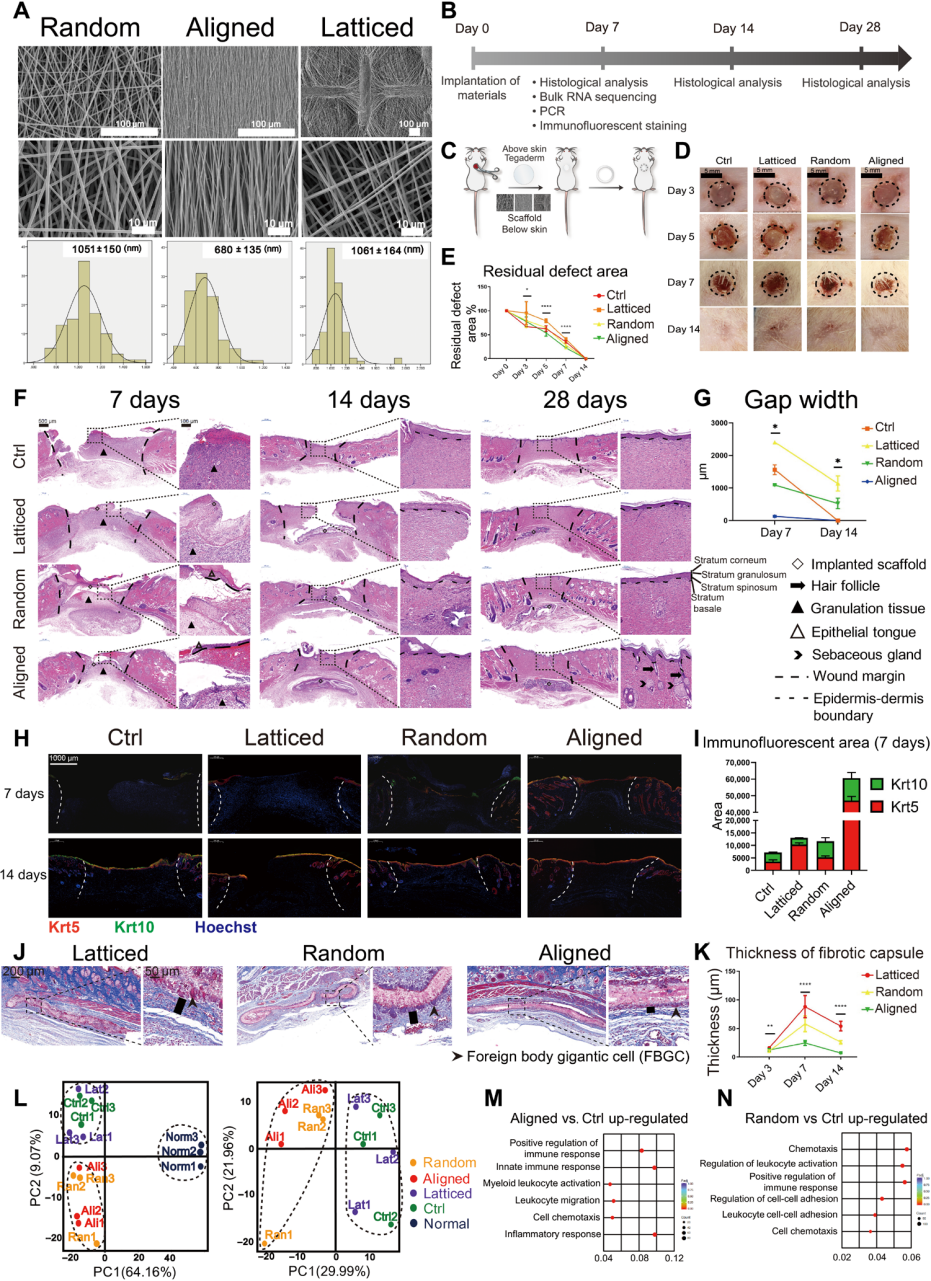
Evaluation of the healing of rat skin wounds implanted with three types of electrospun scaffolds. (**A**) Surface topography of random, aligned, and latticed electrospun membranes. (**B**) Workflow for evaluating rat skin wound healing. PCR, polymerase chain reaction. (**C**) Surgical processes for the rat skin excisional wound model. (**D** and **E**) Residual wound area at 3, 5, 7, and 14 days. Photo credit: Chen Hu, Sichuan University. (**F**) Histological view of the Control (Ctrl) and groups implanted with three types of scaffolds. Regenerated epithelium showed stratified structure at 14 days. (**G**) Semiquantitative evaluation of gap width. (**H**) Immunofluorescent staining using Krt5 (red) and Krt10 (green). (**I**) Semiquantitative evaluation of the fluorescent area. (**J**) Evaluation of foreign body reaction around the biomaterials. Foreign body gigantic cells lined up on the random and latticed membranes, whereas fewer of them appeared on aligned membranes. (**K**) Thickness of fibrotic capsules around random, aligned, and latticed membranes. (**L** to **N**) Principle components (PC) analysis and GO analysis showing immunomodulatory effects for aligned and random scaffolds. ****P < 0.0001, **P < 0.01, and *P < 0.05 by analysis of variance (ANOVA) for data in (E), (G), and (K).

We fabricated scaffolds with random, aligned, and latticed fiber patterns (Fig. 1A) and placed them below the full-thickness excisional wound (diameter, 6 mm) on the rats’ dorsal skin (Sprague-Dawley rat). The Ctrl group received no scaffolds (Fig. 1, B and C). The biophysical properties of the scaffolds are summarized in fig. S1. The workflow for evaluating wound healing is summarized in Fig. 1B. The wound healing rate was substantially accelerated by aligned membranes (Fig. 1, D and E). The latticed group showed delayed wound healing and had the largest residual wound area left on day 7. By day 14, all groups had achieved complete wound closure. Above each scaffold, the surrounding epithelium formed an epithelial tongue as the first layer advanced toward the wound (*16*). On day 7, the aligned group presented the fastest coverage of the wound, leaving the smallest gap width, whereas the latticed membrane seemed to impede the advancement of the surrounding epithelium (Fig. 1, F and G, and fig. S2). On day 14, reepithelialization was complete in all groups except for the latticed group; all groups showed matured stratified epithelia (Fig. 1F). Immunofluorescent staining for Krt5 (keratin secreted by keratinocytes in the basal layer) and Krt10 (keratin secreted by differentiated keratinocytes in the suprabasal layers) showed that, on day 7, the aligned group had the largest area of newly formed keratinized epithelium and more keratinocytes were undifferentiated (Krt5 positive). On day 14, all groups revealed the formation of stratified epithelia (Fig. 1, H and I) (*16*). The dermis, the wound space below the epithelium, was filled with granulation tissue on day 7. Collagen was continuously deposited and remodeled (fig. S2). On day 28, the aligned group had more regenerated hair follicles and sebaceous glands than other treatment groups (Fig. 1F). The scaffolds were placed subcutaneously in rats to evaluate host response against them. The thickness of the fibrotic capsules around scaffolds was measured on days 3, 7, and 14, and the aligned group had the smallest fibrotic capsule thickness at all time points (Fig. 1, J and K, and fig. S3). Bulk-tissue RNA-seq analysis for samples harvested on day 7 (*n* = 3 for each group) identified transcripts corresponding to 34,459 genes, distributed over six orders of magnitude of expression levels. Principal components analysis of the data revealed that the random and aligned samples had more similar gene expression profiles with each other than with the latticed and Ctrl samples (Fig. 1L). Gene ontology (GO) analysis showed that random and aligned scaffolds induced the up-regulation of genes associated with immune responses when compared with the Ctrl samples, whereas the latticed group did not (Fig. 1, M and N). Kyoto Encyclopedia of Genes and Genomes (KEGG) analysis also revealed gene enrichment in an immune-related pathway, “cytokine-cytokine receptor interaction” (KEGG ID rno04060; fig. S4, A and B) (*17*). Real-time quantitative polymerase chain reaction (qPCR) confirmed the elevation of these genes in the aligned and random groups (fig. S4C). To validate that surface topography was a more influential parameter in guiding the differential immune responses, we also applied nondegradable polyvinylidene fluoride (PVDF)–FC scaffolds with latticed, random, and aligned morphology to the mouse cutaneous wound model (fig. S5, A and B). Likewise, the random and aligned PVDF-FC membranes elicited more substantial immune responses than latticed membranes (fig. S5C). To explore whether the scaffolds had a similar performance in other species, we used them in a mouse skin wound model.

### Wound healing in mice

**Fig. 2:**
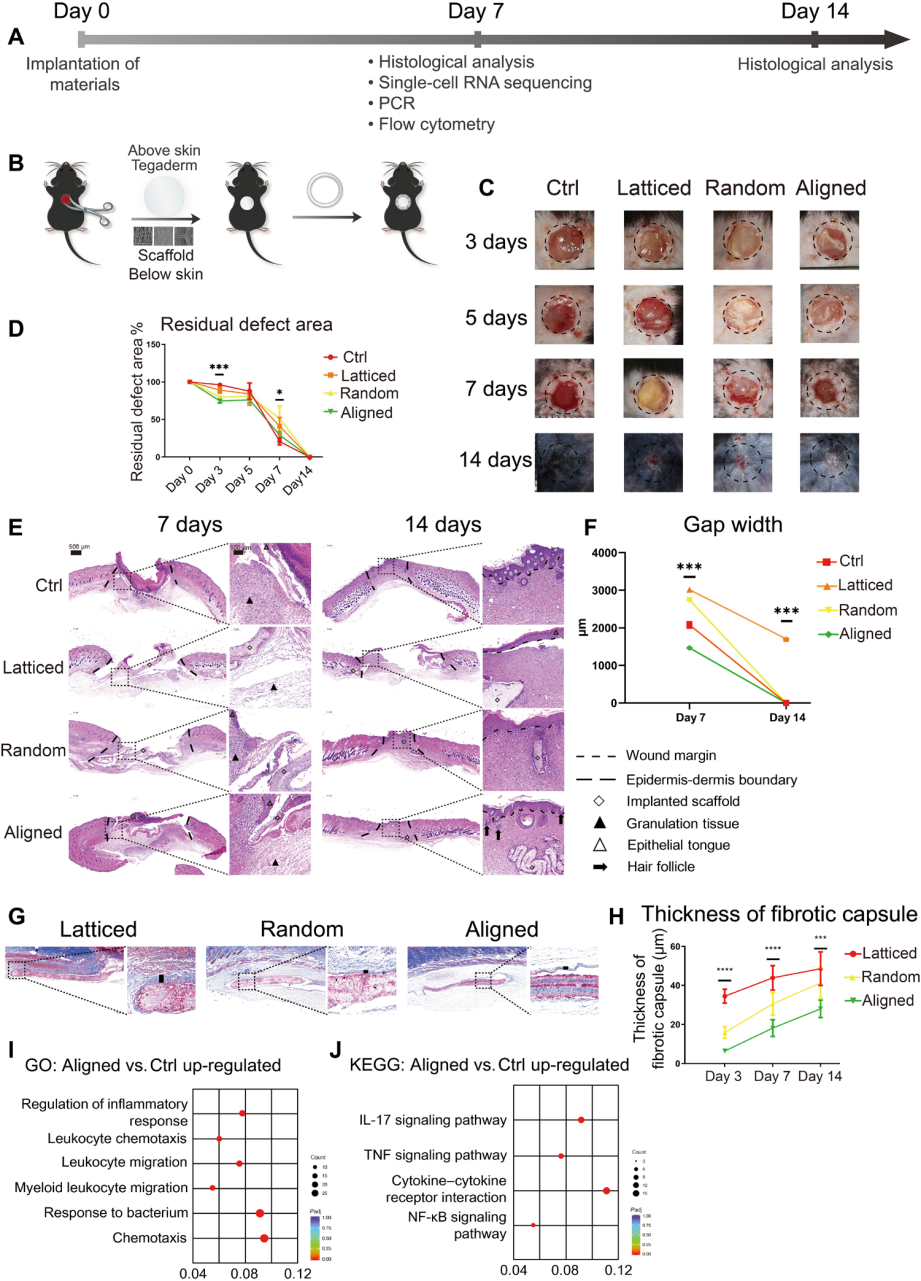
Evaluation of the healing of mouse skin wounds implanted with three types of electrospun scaffolds. (**A**) Workflow for evaluating mouse skin wound healing. (**B**) Surgical processes for mouse skin excisional wound model. (**C** and **D**) Residual wound area at 3, 5, 7, and 14 days. Photo credit: Chenbing Wang, Sichuan University. (**E**) Histological analysis of Ctrl and other groups implanted with three types of scaffolds. (**F**) Semiquantitative evaluation of gap width. (**G**) Evaluation of foreign body reaction around biomaterials. (**H**) Thickness of fibrotic capsules around random, aligned, and latticed membranes. (**I** and **J**) GO and KEGG analysis, respectively, showing immunomodulatory effects for aligned scaffolds. ****P < 0.0001, ***P < 0.001, and *P < 0.05 by ANOVA for data in (D), (F), and (H). NF-κB, nuclear factor κB; TNF, tumor necrosis factor.

Workflow for evaluating wound healing in C57BL/6 mice was summarized in Fig. 2A. We placed three types of scaffolds below the full-thickness excisional wound (diameter, 6 mm; Fig. 2B). The Ctrl group received no scaffolds. Wound coverage was faster in the Ctrl (20.80 ± 4.66%) and aligned (residual wound area, 29.29 ± 4.81%) groups on day 7. On day 14, all groups had achieved complete wound closure (Fig. 2, C and D). The aligned group had the smallest gap width on day 7, followed by the Ctrl group (Fig. 2, E and F). On day 14, reepithelialization was completed in all groups except for some samples in the latticed group. All groups showed mature, stratified epithelia. Hair follicle regeneration was observed in aligned samples after 14 days of healing (Fig. 2E). Scaffolds were also placed subcutaneously to evaluate the host response against them. The aligned membranes had the smallest capsule thickness throughout the observation period (Fig. 2, G and H, and fig. S6). Bulk-tissue RNA-seq was performed for aligned and Ctrl samples harvested on day 7 (*n* = 3 for each group). Compared to the Ctrl, the aligned group showed increased enrichment of genes involved in the inflammatory response, leukocyte chemotaxis, and migration (Fig. 2I). KEGG analysis revealed elevated gene expression in immune-related signaling pathways for the aligned group (KEGG IDs mmu04657, mmu04668, mmu04060, and mmu04064; Fig. 2J). In summary, in both rat and mouse models, the aligned membranes led to faster wound healing, reduced fibrotic response, and enhanced regeneration of cutaneous appendages compared to other membranes. Meanwhile, the aligned scaffold showed immunomodulatory properties. We, therefore, sought to explore how aligned membranes regulated the peri-implant microenvironment. Hence, we used scRNA-seq to sequence cells from the wounded murine full-thickness skin 7 days after wounding.

### Single-cell transcriptome analysis of full-thickness skin after wounding and scaffold placement

**Fig. 3:**
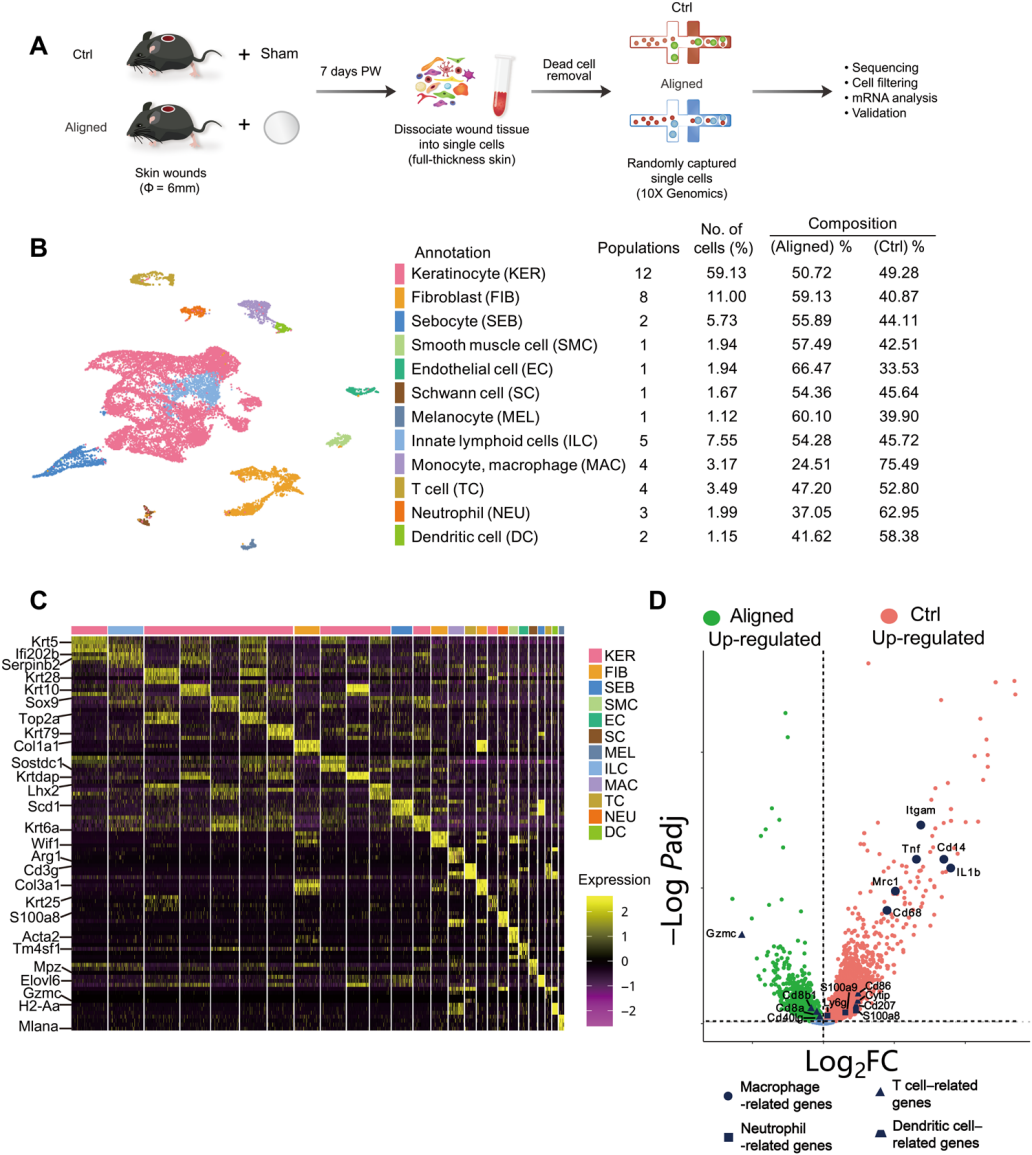
Overview of the single-cell transcriptome analysis. (**A**) Workflow for single-cell experiments. PW, post-wounding. (**B**) Cells from Ctrl and aligned samples are categorized into 12 main classes. Cell populations in each class, number of cells (%), and composition of aligned and Ctrl cells are listed. (**C**) Marker genes for the 26 cell clusters (12 classes). (**D**) Differences in gene expression between Ctrl and aligned groups. log_2_FC, log_2_ fold change.

We isolated cells from the aligned and Ctrl samples (*n* = 4 biological replicates in each group) and applied them to the 10x scRNA-seq platform (Fig. 3A). A total of 8982 cells in the Ctrl group and 9593 cells in the aligned group were captured. After cell filtering, 17,181 single-cell transcriptomes were included in the final dataset (8869 for the aligned group and 8312 for the Ctrl group) (fig. S7). We first computationally pooled cells from Ctrl and aligned groups to create a virtual aggregate. Unsupervised clustering using Seurat categorized the cells into 26 clusters based on global gene expression patterns (fig. S8), which were assigned to 12 main classes of cells (Fig. 3B): keratinocytes (KER), fibroblasts (FIB), sebocytes (SEB), smooth muscle cells (SMC), endothelial cells (EC), Schwann cells (SC), melanocytes (MEL), innate lymphoid cells (ILC), monocyte-macrophages (MAC), T cells (TC), neutrophils (NEU), and dendritic cells (DCs). Marker genes for each cell cluster were shown in the heatmap and listed in fig. S8 (Fig. 3C). The composition of each cell cluster was listed so that the proportion of cells from two groups could be identified across all cell clusters (Fig. 3B). As smooth muscle cells (57% from the aligned group and 43% from the Ctrl group) could hardly regenerate at 7 days after wounding, we regarded a proportion within 50 ± 7% as equilibrium between two groups. Genes related to macrophages (*Itgam*, *Cd68*, *Arg1*, and *Mrc1*) were substantially higher expressed by the Ctrl samples (Fig. 3D).

### Subclustering of keratinocytes reveals a higher proportion of hair follicle progenitor cells from the aligned group

**Fig. 4:**
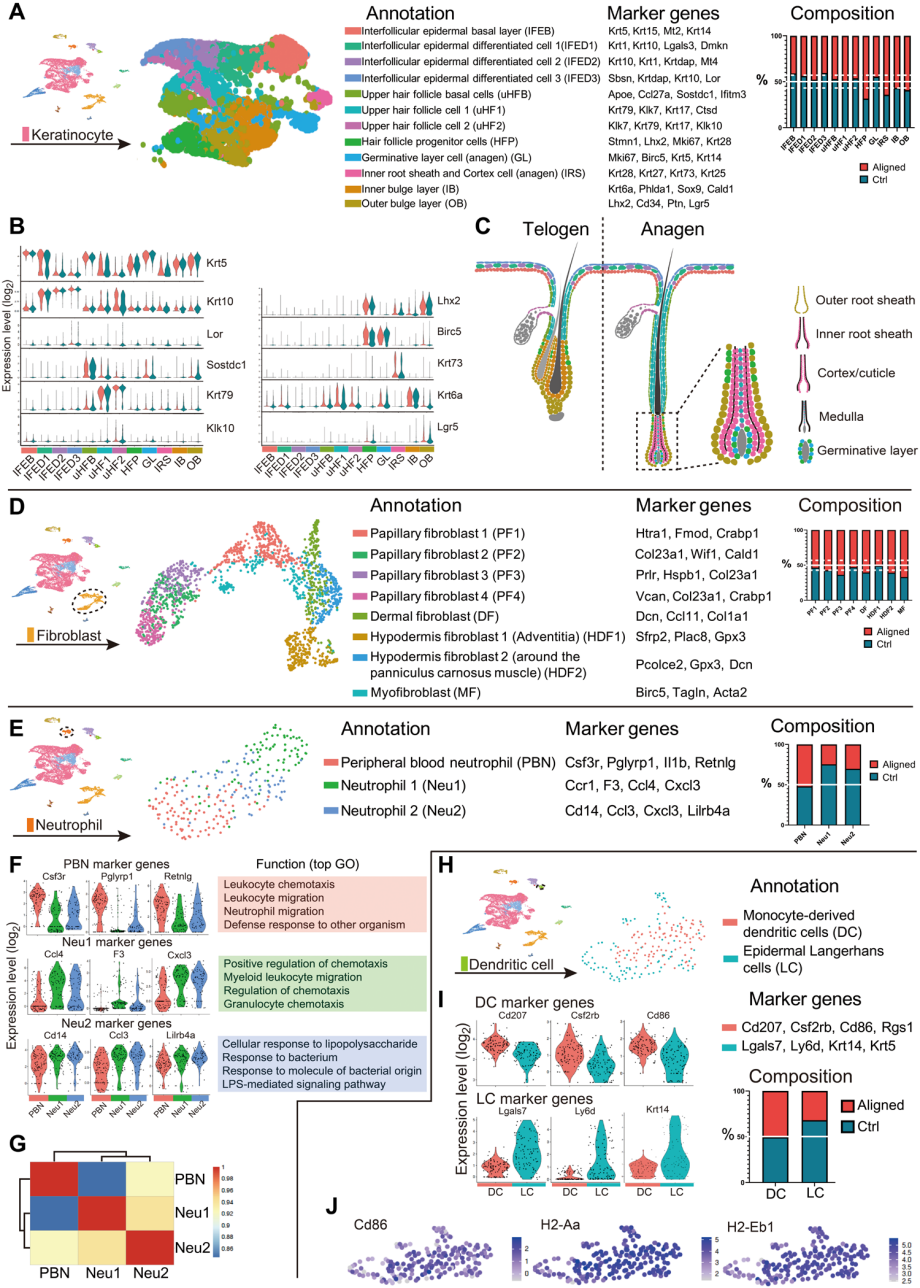
Further analysis of keratinocytes, fibroblasts, neutrophils, and DCs. (**A**) Subclustering of keratinocytes showing 12 subsets. The marker genes and composition for each subset are listed. (**B**) Marker genes for each keratinocyte subset. (**C**) Location of different keratinocyte subset. Color of each cell corresponds with its cell type in (A). (**D**) Subclustering of fibroblasts showing eight subsets. The marker genes and composition for each subset are listed. (**E**) Subclustering of neutrophils showing three subsets. (**F**) Marker genes for neutrophil subsets and their enriched gene sets in GO analysis. LPS, lipopolysaccharide. (**G**) Correlation analysis of neutrophil subsets. (**H**) Subclustering of DCs showing two subsets. (**I**) Marker genes for DC subsets. (**J**) Expression of genes associated with antigen presenting in DCs.

We selected cells that were in the first-level clustering defined as keratinocyte (fig. S8) and subjected them to a second round of unsupervised clustering (Fig. 4A). Terminal cornification of epidermis is achieved by keratinocytes passing through basal layers, differentiated layers, and cornified layers (*16*). In our study, the interfollicular epithelium was composed of the basal layer cells [IFEB (interfollicular epidermal basal layer) and *Krt5*^hi^*Krt14*^hi^], suprabasal layer cells [*Krt10*^hi^*Krt1*^hi^ interfollicular epidermal differentiated cell 1 (IFED1) and *Krt10*^hi^*Mt4*^hi^ IFED2], and cornified layer (*Lor*^+^ IFED3) cells (Fig. 4, A and B). The location of keratinocyte subsets was marked in Fig. 4C. Hair follicles in this study were in second telogen because the mice were 8 to 10 weeks old when they were euthanized (*18*). However, anagen hair follicle gene signatures were also found, possibly resulting from hair follicle regeneration after wounding. The upper hair follicle cells were separated into three subsets named uHF1 (upper hair follicle cell 1) (*Krt79*^hi^*Krt17*^hi^), uHF2 (*Klk10*^+^*Krt79*^hi^), and uHFB (the upper hair follicle basal layer cells, *Sostdc1*^hi^*Apoe*^hi^). The hair follicle progenitor (HFP) cells (*Krt28*^hi^*Lhx2*^hi^*Mki67*^hi^ HFP) were highly proliferative. Germinative layer cells (*Mt2*^hi^*Dcn*^hi^) belonging to anagen hair follicles also expressed high cell proliferation–related genes like *Top2a* and *Birc5*. The inner root sheath (IRS) and cortex cells were characterized by *Krt28*, *Krt27*, *Krt73*, and *Krt25*, markers for the Henle and Huxley layers of anagen hair follicles (*19*). Cells of the inner bulge layer (IB) and outer bulge layer expressed their typical gene signatures (Fig. 4, A to C). When analyzing the intergroup differences, the aligned group contributed to a larger proportion of HFP cells and highly proliferative IRS cells (Fig. 4A). This might explain the enhanced hair follicle regeneration observed in aligned groups either on rat or mouse models.

### Intergroup differences in fibroblasts suggest more active extracellular matrix formation in the aligned group

The dermis consists of several layers: the papillary dermis that lies closest to the epidermis, the underlying reticular dermis containing the bulk of the fibrillary extracellular matrix, and the hypodermis that lies beneath the reticular dermis (*20*). Fibroblasts from different layers presented distinct gene signatures (Fig. 4D). There were four populations of papillary fibroblasts (*Crabp1*^+^*Col23a1*^+^) (*21*, *22*). Dermal fibroblasts expressed increased *Ccl11* and *Dcn*. The *Gpx3*^+^*Plac8*^hi^ subset was identified as the hypodermal fibroblast located close to the adventitia [hypodermis fibroblast 1 (HDF1)], and the *Gpx3*^+^*Plac8*^lo^ subset (HDF2) was around the panniculus carnosus muscle (*19*). Contractile myofibroblasts (*Acta2*^+^ MF) expressed elevated genes associated with cell proliferation (*Birc5*^hi^). The composition of each subset revealed that more papillary [PF2 (papillary fibroblast 2)], dermal [DF (dermal fibroblast)], hypodermal (HDF2), and MF were observed in the aligned group, suggesting more robust extracellular matrix formation in the presence of scaffolds.

### Neutrophils and DCs were more abundant in the Ctrl samples

After wounding, neutrophils close to the center of the injury migrated toward the nidus, followed by those recruited more than 200 μm from the site of tissue injury (*23*). Three neutrophil subsets were identified in our study (Fig. 4E). Peripheral blood neutrophils (PBNs) expressed typical gene signatures, including *Csf3r*, *Pglyrp1*, *Il1b*, and *Retnlg*. Neutrophil 2 (Neu2) expressed elevated *Cd14* and *Ccl3* and was identified as antimicrobial phagocytic neutrophils. Neu1 expressed elevated *Ccr1*, a chemokine receptor that mediates neutrophil migration. Correspondingly, PBNs showed gene enrichment in leukocyte chemotaxis and migration; Neu1 expressed genes enriched in leukocyte/granulocyte chemotaxis and migration, and Neu2 was enriched in antibacterial biological processes (Fig. 4F). According to the correlation analysis, Neu 1 and Neu 2 were derived from PBNs in circulation (Fig. 4G). In Neu1 and Neu2, more cells belonged to the Ctrl group (Fig. 4E), suggesting more substantial Neu1 and Neu2 infiltration in the Ctrl samples at the proliferative stage (7 days after wounding). DCs were classified into two subpopulations (Fig. 4H). The subset derived from monocytes (DC) was characterized by increased *Cd207* and *Cd86* (*11*, *24*). The Langerhans cell (LC) subset bore a keratinocyte gene signature, probably transferred from the resident microenvironment (Fig. 4I) (*25*). Both DC and LC presented elevated major histocompatibility complex (MHC) molecules, suggesting they had an antigen-presenting function (Fig. 4J). LCs contained more Ctrl-derived cells, indicating differences in LC infiltration between groups.

### Macrophage heterogeneity and their down-regulation by scaffolds

**Fig. 5:**
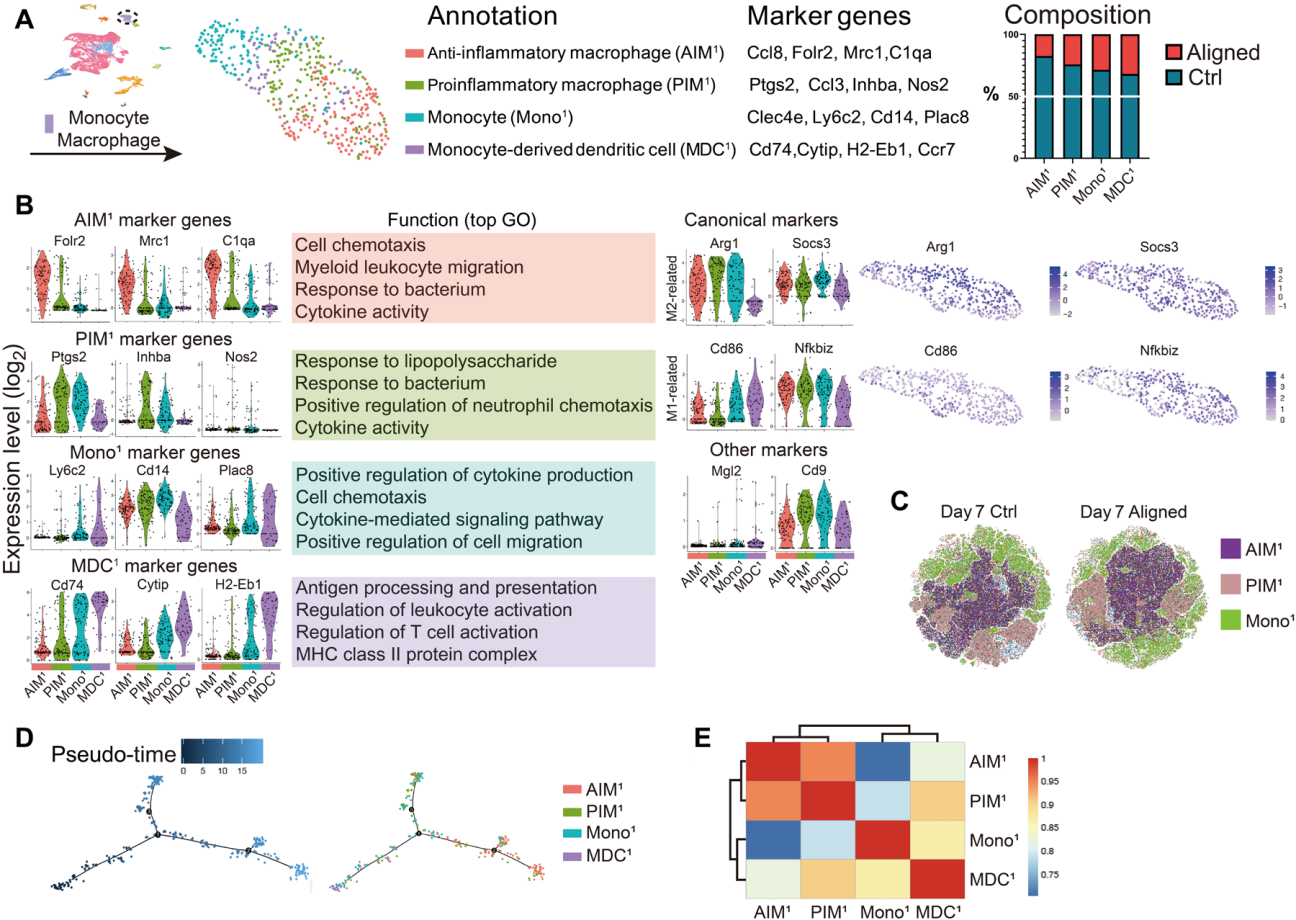
Analysis of macrophages. (**A**) Subclustering of macrophages showing four subsets. The marker genes and composition for each subset are listed. (**B**) Marker genes for each macrophage/monocyte subset and their enriched gene sets in GO analysis. Expression of canonical M1 and M2 markers and other proposed markers is shown. (**C**) In vivo flow cytometry strategy using CD68 to identify monocytes and macrophages. ARG1^+^ macrophages expressing the surface markers MRC1 and NOS2 in the gated dataset represent anti-inflammatory macrophages (AIM^1^) and proinflammatory macrophages (PIM^1^), respectively. The ARG1^+^LY6C^+^ monocytes (Mono^1^) are also shown. (**D**) Pseudo-temporal ordering of macrophage/monocytes and the distribution of four subsets along the trajectory. (**E**) Correlation analysis of macrophage/monocyte subsets.

To explore the heterogeneity of macrophages in vivo and their intergroup differences, we subjected them to further unsupervised subclustering. Four subsets were determined (Fig. 5A). The subset that showed increased anti-inflammatory genes (*Ccl8*, *Folr2*, *C1qa*, and *Mrc1*) was named anti-inflammatory macrophages (AIM^1^) (*26*–*28*). Another subset characterized by elevated expression of proinflammatory genes (*Ptgs2*, *Ccl3*, *Inhba*, and *Nos2*) was named proinflammatory macrophages (PIM^1^) (*29*, *30*). The monocyte (Mono^1^) subset showed higher *Ly6c2*, *Plac8*, *Cd14*, and *Clec4e*, indicating inflammatory responses against lesions and microorganisms (*31*, *32*). The *Cytip*^hi^*H2-Eb1*^hi^ cell subset was defined as monocyte-derived DCs (MDC^1^; Fig. 5B) (*33*). We further found that canonical M1 and M2 markers were not entirely consistent with computationally determined AIM^1^ and PIM^1^. *Arg1*, a canonical M2 marker, was expressed by all monocyte-macrophage subsets (AIM^1^, PIM^1^, and Mono^1^) and regarded as a pan-macrophage marker in this study (Fig. 5B). The expression of another type 2 gene, *Socs3*, did not also parallel *Mrc1* expression, and a similar disparity was found in the expression of canonical type 1 genes. MDC^1^, rather than PIM^1^, expressed more *Cd86*, and *Nfkbiz* did not correlate with Cd86. The expression of other genes associated with fibrotic or regenerative macrophage subsets in a scaffold immune microenvironment did not correspond with these clusters (Fig. 5B) (*13*). *Ly6c2*, *Arg1*, *Mrc1*, and *Nos2* were determined to sufficiently distinguish the computationally determined AIM^1^, PIM^1^ and Mono^1^ subsets. We performed flow cytometry on cells isolated from aligned and Ctrl samples using CD68, a monocyte-macrophage marker also expressed by some neutrophils and DCs, and the proposed markers (*Ly6c2*, *Arg1*, *Mrc1*, and *Nos2*). CD68^+^ cells were selected to create a *t*-distributed stochastic neighbor embedding (*t*SNE) plot. We then identified ARG1^+^ macrophages expressing the surface markers, MRC1 and NOS2, in the gated dataset to represent AIM^1^ and PIM^1^, respectively. The ARG1^+^LY6C^+^ Mono^1^ were also identified. The three terminal clusters (AIM^1^, PIM^1^, and Mono^1^) could be separated, indicating that the subsets could be identified experimentally using flow cytometry (Fig. 5C). The pseudo-temporal trajectory (Monocle 2) of the four subsets revealed that monocytes developed into MDC^1^ and polarized macrophages (AIM^1^ and PIM^1^). Although AIM^1^ and PIM^1^ expressed distinct gene signatures, they were highly correlated (Fig. 5, D and E). The Ctrl samples contributed to a larger proportion of cells in all subsets (Fig. 5A). Therefore, the scaffold might play a role in the reduction of macrophage infiltration at the proliferative stage.

### Subclustering of T cells revealed a previously unidentified T cell population and more effector T cells in the aligned group

**Fig. 6:**
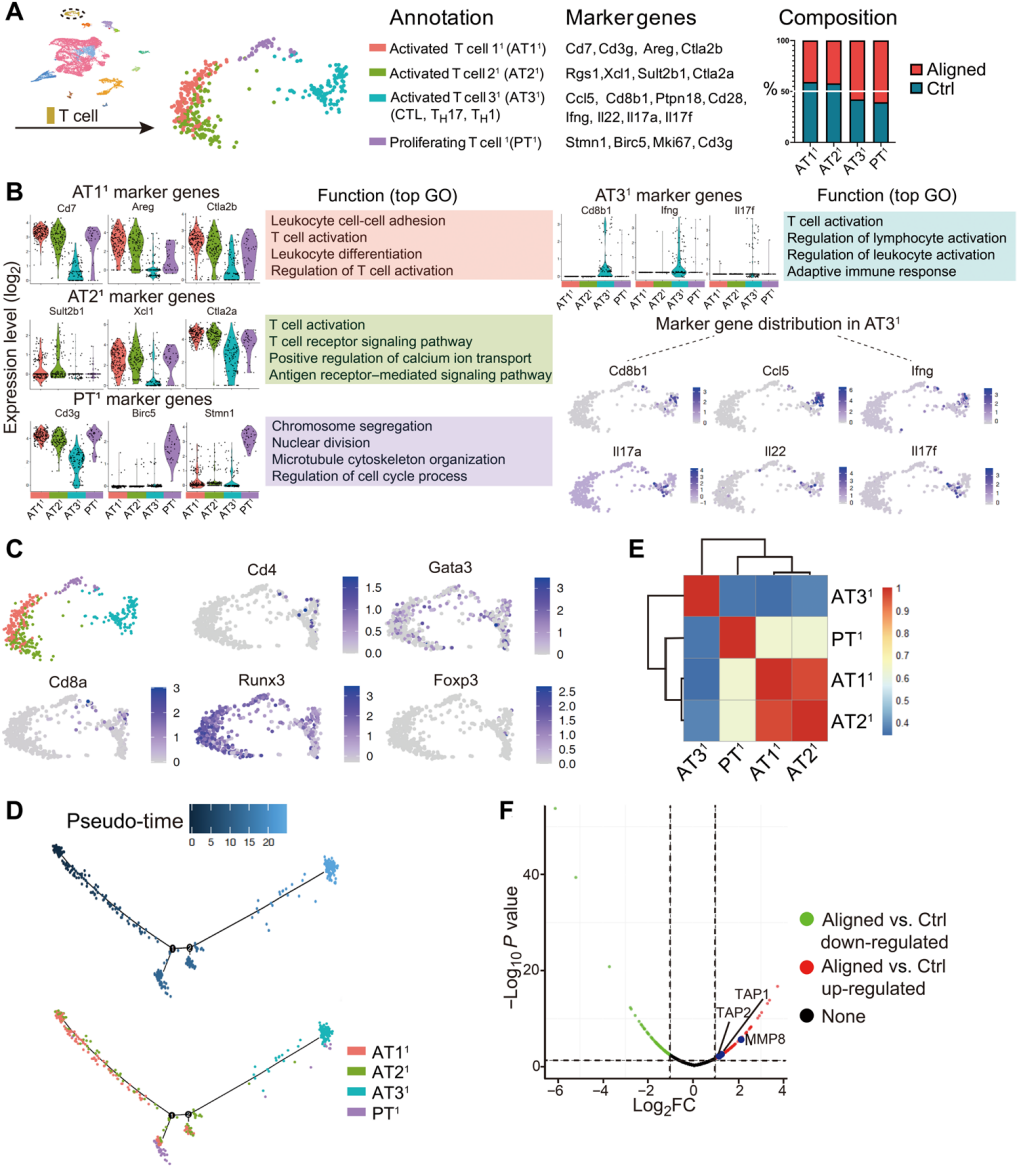
Analysis of T cells. (**A**) Subclustering of T cells showing four subsets. (**B**) Marker genes for T cell subsets and their enriched gene sets in GO analysis, showing expression of the gene signatures of cytotoxic T cells, T_H_1 cells, and T_H_17 cells by the AT3^1^ subset. (**C**) Expression of canonical T cell markers are inconsistent with the current computer-derived clustering. (**D**) Pseudo-temporal ordering of T cells and the distribution of four subsets along the trajectory. (**E**) Correlation analysis of T cell subsets. (**F**) Proteomic assay for Ctrl and aligned groups showed higher Tap expression by aligned samples.

T cells were clustered into four subsets (Fig. 6A). Those characterized by increased *Cd7*, *Cd3g*, and *Areg* genes were named Activated T cell 1^1^ (AT1^1^) (*34*). The subset adjacent to AT1^1^ expressed elevated *Xcl1* and *Sult2b1* genes (associated with T cell activation) and was named Activated T cell 2^1^ (AT2^1^) (*35*). AT2^1^ also expressed increased *Areg*, *Ctla2a*, and *Ctla2b*, genes related to immune homeostasis and immunosuppression (*36*, *37*). Another activated T cell subset (AT3^1^) expressed up-regulated genes associated with cytotoxic T cells (*Cd8b1*), T_H_1 cells (*Ifng* and *Ptpn18*), and T_H_17 cells (*Il17a* and *Il17f*) (*38*, *39*). Therefore, AT3^1^ might include multiple effector T cells. A T cell subset connecting AT3^1^ and AT1^1^ was characterized with high expression of *Birc5*, *Mki67*, and *Stmn1* markers for cell proliferation and was named Proliferating T cell^1^ (PT^1^) (Fig. 6, A and B). GO analysis showed that both AT1^1^ and AT2^1^ were enriched in T cell activation. Further, the T cell receptor signaling pathway was elevated in AT2^1^, suggesting that these T cells played a role in antigen recognition (Fig. 6B). Gene expression of canonical T cell markers and typical transcription factors (*Cd4*, *Cd8a*, *Foxp3*, *Gata3*, and *Runx3*) were not entirely consistent with the computationally determined subsets (Fig. 6C), suggesting diverse T cell heterogeneity in vivo. Pseudo-time analysis revealed three terminally differentiated clusters stemming from two precursors (AT1^1^ and AT2^1^). PT^1^ was differentiated from AT1^1^ and AT2^1^ after the first branch point and might be a transitional status between early and effector T cells. At the second branch, cells differentiated into two terminal clusters; one belonged to AT1^1^ and AT2^1^, and the other, to the AT3^1^ population (Fig. 6D). Correlation analysis showed that AT1^1^ and AT2^1^ were highly correlated (Fig. 6E). More cells in the AT1^1^ and AT2^1^ populations were from the Ctrl samples, whereas a larger number of effector T cells and PT^1^ were from the aligned samples. Correspondingly, proteomics assay for the aligned and Ctrl samples revealed that the aligned group expressed higher TAP1 and TAP2 (Fig. 6F), transporters associated with antigen processing (*40*). GO enrichment showed protein enrichment in MHC protein binding (*P* = 0.0059) and TAP (transporter associated with antigen processing) complex (*P* = 0.0059) for the aligned sample. InterPro analysis also indicated enrichment of TAP in the aligned group (*P* = 0.00205). The higher TAP expression level confirmed elevated T cell activities in aligned samples.

### Receptor-ligand analysis reveals intricate interactions among immune cells, keratinocytes, and fibroblasts

**Fig. 7:**
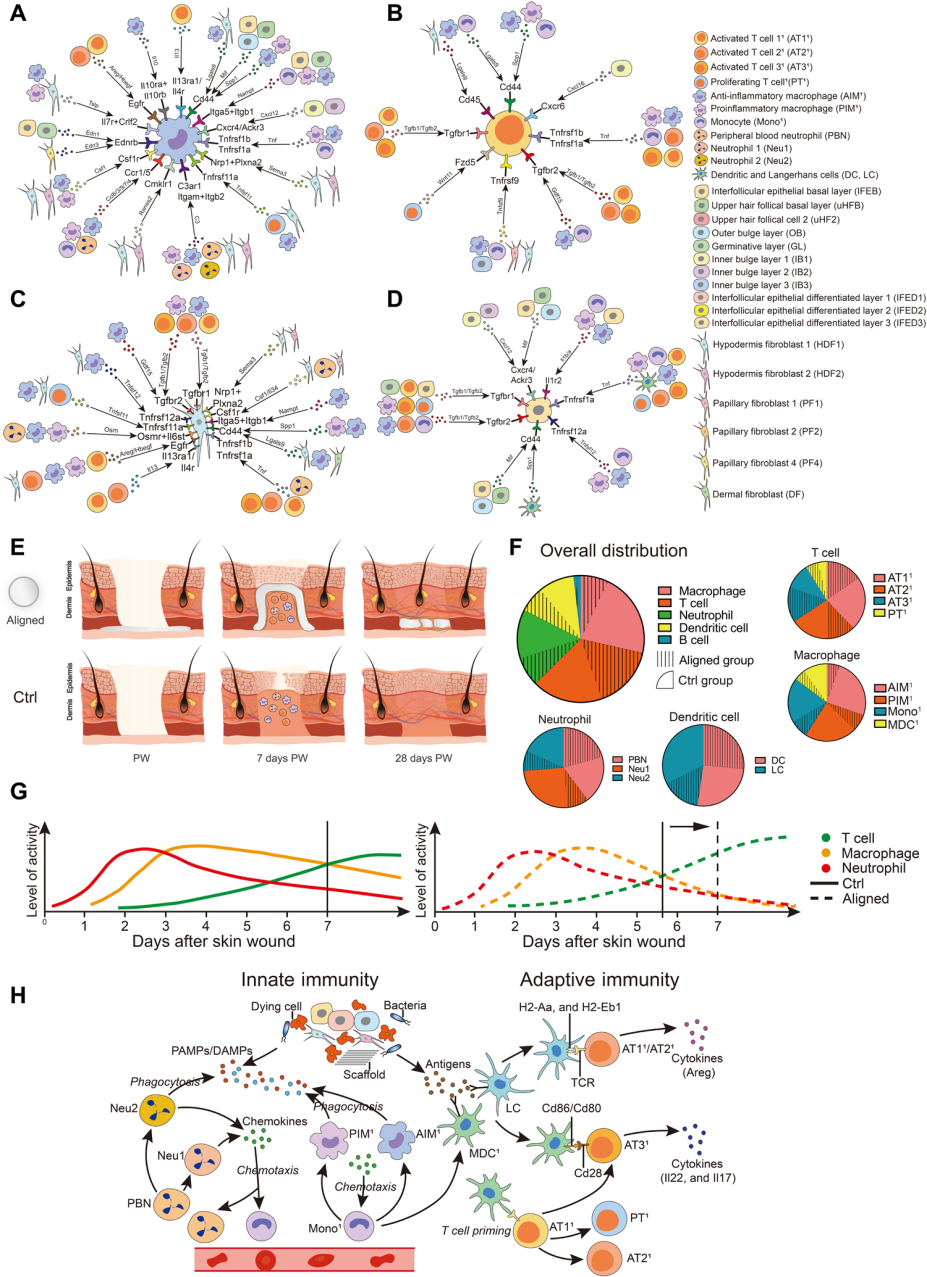
Cell-cell interaction plots according to receptor-ligand analysis using CellChat, as well as summary of the differences in wound healing and immune responses between the aligned and control (Ctrl) groups. (**A**) Signals between AIM^1^ and other cell types. (**B**) Signals between AT1^1^ and other cell types. (**C**) Signals between HDF1 and other cell types. (**D**) Signals between IFEB and other cell types. (**E**) The aligned membranes had immunomodulatory properties and led to improved healing. (**F**) Pie plots of the immune cells in the aligned and Ctrl groups showing differences in cell composition in each cell type. (**G**) Time frame of innate and adaptive immune responses in the aligned and Ctrl groups. (**H**) Immune microenvironment around aligned scaffolds.

To explore potential interactions among immune cells, keratinocytes, and fibroblasts, we performed CellChat analysis on these datasets (*41*). For AIM^1^, broadly speaking, “proinflammatory” signals, including Tnf, Visfatin, Rankl, epidermal growth factor receptor, and C3a, and “anti-inflammatory” signals, including Spp1, Lgals9, Sema3, Chemerin, Il2, Il13, and Il10, were involved via either the paracrine or autocrine system. Mif, Ccl, Csf, Edn, and Cxcl12 signaling exerted protective or deleterious effects depending on their specific roles (Fig. 7A) (*26*, *42*–*53*). Among T cell populations (Fig. 7B), AT1^1^ communicated with other T cells mainly through Tgfb signaling, mediating T cell tolerance, and immune homeostasis (*54*). The PT^1^ population secreted Wnt11 signals that bound to the Fzd5 receptor on AT1^1^. The IBs secreted Cxcl16 that targeted Cxcr6 on AT1^1^, chemoattracting AT1^1^ to the wound area (*55*). For fibroblast populations, HDF1 had the most frequent interaction with other cells (Fig. 7C). In addition to the mentioned signals, oncostatin M signaling was also activated and induced the expression of the proinflammatory cytokine IL-6 (*56*). T cells and macrophages secreted Tgfb1 and Tgfb2 to target HDF1 and PF1, leading to the proliferation and differentiation of fibroblasts into myofibroblasts (*57*). Among keratinocytes, IFEB and IB1 had the most frequent contact with other cells (Fig. 7D and fig. S9, I to M). IFEB received Tgfb1 and Tgfb2—signals playing key roles in regulating epithelial-to-mesenchymal transition for keratinocytes—from T cells, macrophages, and germinative layer keratinocytes (*58*). Interactive plots for other cell types are summarized in fig. S9. The communication between immune and cutaneous cells was complicated, and wound healing resulted from numerous influencing factors working together.

### Differences in immune microenvironments around random, latticed, and aligned scaffolds and a regulatory role of T cells in the wound healing process

**Fig. 8:**
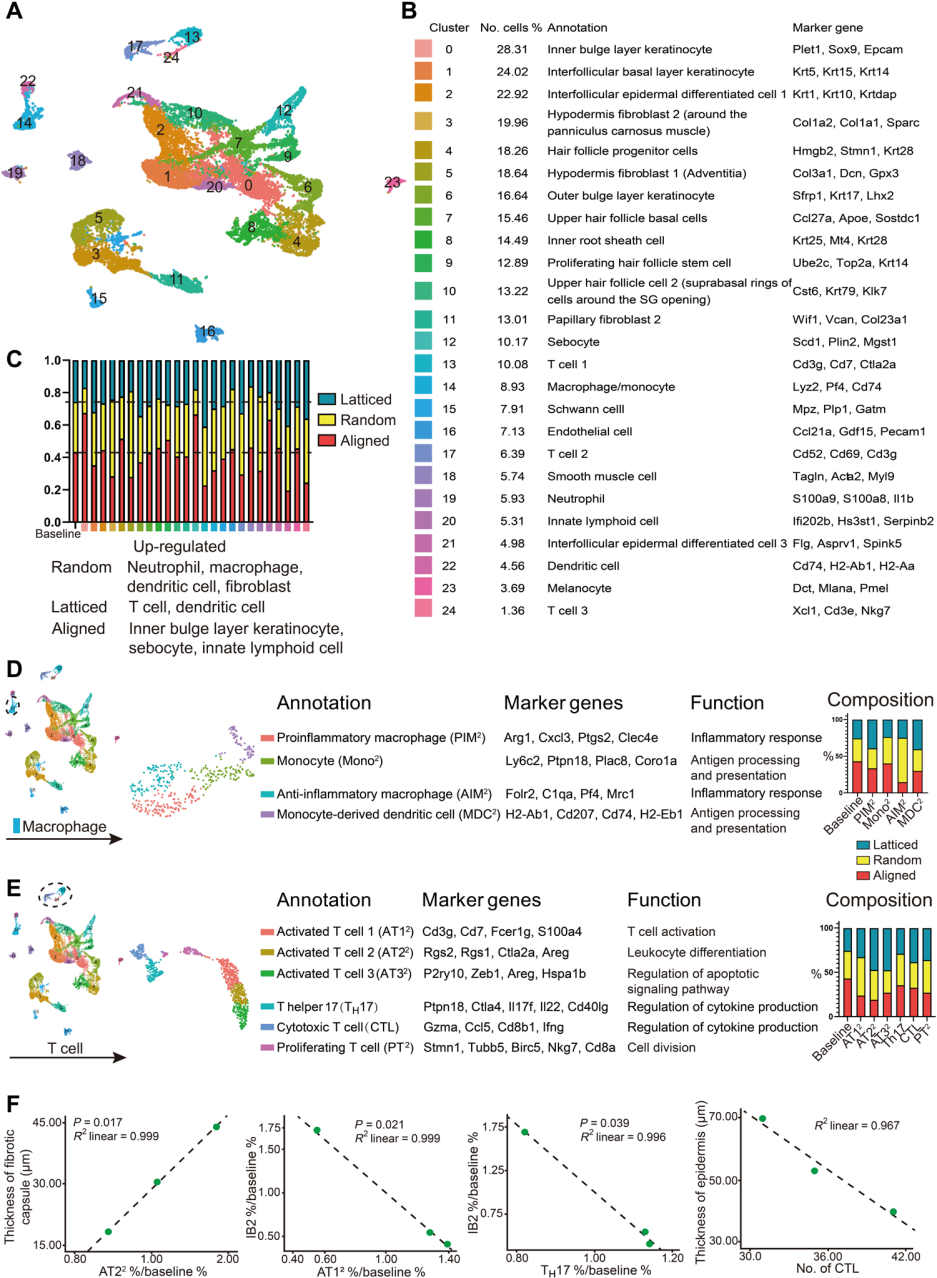
Integrated analysis of three scaffolds showing differences in immune microenvironments around random, latticed, and aligned scaffolds. (**A**) Cells from random, aligned, and latticed samples are clustered into 25 clusters. (**B**) Number of cells (%) in each cluster and their marker genes are listed. (**C**) Composition of cells in each cluster. (**D**) Subclustering of macrophages showing four subsets. The intergroup differences are listed. (**E**) Subclustering of T cells showing six subsets. The intergroup differences are listed. (**F**) Correlations found between T cells and healing parameters. IB2 refers to Ifi202b^+^Plet1^+^Plk2^+^Krt6a^+^ inner bulge layer keratinocytes.

We further explored the differences in cell composition and gene expression among random, latticed, and aligned scaffolds using scRNA-seq. Cells from three groups were computationally pooled to create a virtual aggregate. Unsupervised clustering using Seurat categorized the cells into 25 clusters based on global gene expression patterns (Fig. 8, A and B). The aligned samples recruited less adaptive immune cells (*Cd3g*^hi^*Cd7*^hi^*Nkg7*^hi^ T cells) than the latticed group and, meanwhile, less innate immune cells (macrophages, neutrophils, and DCs) compared to the random group (Fig. 8C). We further explored the differently distributed immune cells. Subclustering of macrophages resulted in four subsets, which were PIM^2^, AIM^2^, Mono^2^, and MDC^2^ (Fig. 8D). Aligned samples that showed improved wound healing had the lowest number of anti-inflammatory macrophages (AIM^2^, *Folr2*^hi^*C1qa*^hi^*Mrc1*^hi^) (*26*, *28*), whereas random samples recruited the most AIM^2^ (Fig. 8D). The latticed group had the most proinflammatory macrophages (PIM^2^, *Arg1*^hi^*Ptgs2*^hi^*Clec4e*^hi^), followed by the aligned group, and the random group showed the lowest PIM^2^ recruitment. The results indicated that random scaffolds induced a more anti-inflammatory microenvironment than aligned and latticed ones did, which did not correspond with the wound healing outcomes. Meanwhile, we found no notable correlation between the number of macrophages (AIM^2^ and PIM^2^) and healing parameters (residual wound area and thickness of fibrotic capsules). Therefore, we considered that anti- and proinflammatory macrophages were not the predominant cell type influencing wound healing at day 7.

**Fig. 9:**
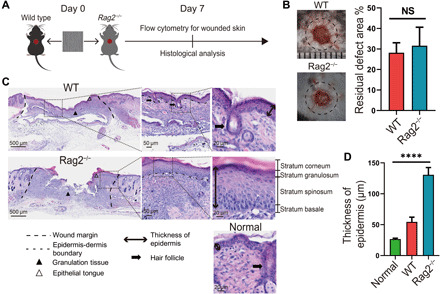
Evaluation of wound healing in *Rag2*^−/−^ mice. (**A**) Workflow for evaluating wound healing in *Rag2*^−/−^ and WT mice. (**B**) Residual defect area at 7 days after wound. Photo credit: Chen Hu, Sichuan University. (**C**) Histological analysis of wound recovery in WT and *Rag2*^−/−^ mice. Normal refers to normal mouse skin. (**D**) Semiquantitative evaluation of thickness of epidermis.

Subclustering of T cells resulted in six subsets. According to their marker genes and gene enrichment analysis, the T cell subsets were named Activated T cell 1^2^ (AT1^2^, *Cd3g*^+^*Cd7*^+^*Fcer1g*^+^), Activated T cell 2^2^ (AT2^2^, *Rgs2*^+^*Ctla2a*^+^*Areg*^+^), Activated T cell 3^2^ (AT3^2^, *P2ry10*^+^*Zeb1*^+^*Hspa1b*^+^), T_H_17 (*Il17f*^+^*Ctla4*^+^*Ptpn18*^+^), cytotoxic T lymphocyte (CTL) (*Gzma*^+^*Cd8b1*^+^*Ifng*^+^), and Proliferating T cell^2^ (PT^2^, *Stmn1*^+^*Birc5*^+^*Tubb5*^+^) (Fig. 8E). Latticed and random samples contributed to more T cells in all subsets. When three scaffolds were analyzed, correlation analysis showed that the amount of AT2^2^ cells was positively correlated with thickness of fibrotic capsules (*P* = 0.017 and *R*^2^ = 0.999). Meanwhile, the amount of T_H_17 cells (*P* = 0.039 and *R*^2^ = 0.996) and AT1^2^ (*P* = 0.021 and *R*^2^ = 0.996) cells was negatively correlated with the amount of IB2 keratinocytes (*Ifi202b*^+^*Plet1*^+^*Plk2*^+^*Krt6a*^+^). The thickness of newly formed epidermis was negatively correlated with number of CTLs (*P* = 0.116 and *R*^2^ = 0.967) (Fig. 8F). On the basis of this, we considered that the amount of T cells might influence healing outcomes including fibrotic response, formation of skin appendages, and structure of regenerated epidermis. To testify that, we placed aligned scaffolds in the dorsal skin wounds of immunodeficient C57Bl/6 (B6.129-Rag2tm1) mice, which lacked mature T lymphocytes (Fig. 9A). We isolated cells of the wounded skin tissue (harvested at 7 days after wound) and confirmed significant CD3^+^ T cell reduction in the *Rag2*^−/−^ mice (fig. S10). Wound closure rate was not significantly different between wild-type (WT) and *Rag2*^−/−^ mice (*Rag2*^−/−^) (Fig. 9B). The new epithelium was much thicker in *Rag2*^−/−^ mice, characterized by hyperplasia of stratum spinosum and stratum corneum. Regeneration of skin appendages was scarce in *Rag2*^−/−^ mice (Fig. 9, C and D).

## DISCUSSION

The skin-wound repair process was classically divided into four phases, namely, hemostasis (hours), inflammation (days), proliferation (1 to 2 weeks), and remodeling (>2 weeks). Seven days after wounding was a transitional time point when the innate immune response subsided and the activity of adaptive immune cells increased (Fig. 7E) (*11*). In the Ctrl samples, the infiltration of T cells occurred to a similar extent as with macrophages (Fig. 7F; overall distribution). However, in the aligned group, infiltrated macrophages were much fewer than T cells, and more terminally differentiated effector T cells were present (Fig. 7F). According to the time frame of innate and adaptive immune responses (*11*), the process of innate immunity seemed to be alleviated earlier, and adaptive immune response was advanced in the presence of aligned scaffolds (Fig. 7, F and G). In the immune microenvironment around aligned scaffolds (Fig. 7H), damage-associated molecular patterns, pathogen-associated molecular patterns, and antigens from cell debris, pathogens, and foreign agents (scaffold) triggered innate and adaptive immune responses. Neutrophils from circulation (PBNs) quickly migrated to the wound area. The infiltrated neutrophils (Neu1 and Neu2) phagocytosed dying cells and microorganisms, and secreted chemoattractants, like Ccr1 and Ccl4, to recruit more leukocytes and lymphoid cells (*55*). Circulating monocytes (Mono^1^) were also recruited to the wound area and differentiated into PIM^1^, clearing dying neutrophils and debris. Meanwhile, AIM^1^ chemoattracted more cells via chemokines, including Ccl8 and Ccl6. Some monocytes differentiated into MDC^1^. Tissue resident LCs, together with MDC^1^, functioned as antigen-presenting cells (APCs). Following antigen-specific signaling, LC and DC processed antigens into peptides and presented them by MHC class II molecules (H2-Aa and H2-Eb1) on the cell surface. T cells (AT1^1^ and AT2^1^) bound to the MHC molecules through surface receptors (T cell receptor) and then differentiated into effector T cells (AT3^1^). The costimulatory signal was characterized by the engagement of the CD28 receptor on T cells (AT3^1^, *Cd8b1*^+^*Cd28*^hi^) with CD86 ligands on APCs (Cd86^+^ DC and LC) (*11*, *59*). The influence of the immune microenvironment on tissue generation was displayed, as immune cells sent out a variety of signals (Grn, Tgfb, and Areg/Hbegf) that modulated the behaviors of keratinocytes and fibroblasts (Fig. 7, A to D). Conversely, keratinocytes and fibroblasts secreted chemotactic pro- or anti-inflammatory signals that regulated immune cell polarization and function. Immune cells also interacted with each other via numerous signals; however, the communication network of immune and cutaneous cells was complicated, and wound healing resulted from the combined effect of these factors.

Wound-induced hair neogenesis (WIHN) usually occurs upon substantial injury such as full-thickness excisional wounding larger than 1 cm^2^, whereas small wounds (<1 cm^2^) typically repair by forming scar tissue (*60*). However, in this study, hair follicles regenerated in the center of a small wound (φ = 6 mm) in the presence of aligned scaffold (7 days after wound) (Fig. 3C). The major difference between aligned and Ctrl samples was the macrophage population and some effector T cells; hence, we focused on the influences of macrophages and T cells on wound healing. Furthermore, when the random, latticed, and aligned groups were analyzed together, the infiltration of macrophages did not correlate with the healing outcomes, whereas T cells had some correlations with the healing parameters. Therefore, we focused on the role of T cells in regulating mouse skin wound healing. By placing aligned scaffolds into *Rag2*^−/−^ mice, we observed that the wound closure was not delayed. The regenerated tissue in *Rag2*^−/−^ mice had no hair follicles and was characterized by notable hyperplasia of stratum spinosum and stratum corneum. As hair follicles did not regenerate both in wounds infiltrated with large amount of T cells (latticed samples) and in *Rag2*^−/−^ mice, we proposed that T cells might play dual roles in regulating WIHN and that proper amount of T cell recruitment was essential for WIHN in small wounds (diameter, 6 mm; circular wound). Future studies should address how T cells regulate hair follicle neogenesis. The epidermal hyperplasia (acanthosis) observed in *Rag2*^−/−^ mice was similar to skin lesions of psoriasis. The pathogenesis of psoriasis was related to the IL-23/T_H_17 pathway involving IL-22, preferentially produced by T_H_17 cells, mediating the acanthosis induced by IL-23 (*61*). Although *Rag2*^−/−^ mice lacked mature T cells, IL-23, IL-22, and IL-17 can also be produced by type 3 innate lymphoid cells, DCs, and macrophages (*39*, *62*); these cells might have compensated for the production of IL-23 and promoted the epidermal hyperplasia. In conclusion, immune responses toward the biomaterials with different scaffolds varied substantially from that of the Ctrl group. The aligned group displayed an adaptive immunity–dominant response and yielded the most desirable results among three scaffolds. Integrated analysis of three scaffolds suggested that T cells had modulatory effects on wound healing. In *Rag2*−/− mice, lack of WIHN and epidermal hyperplasia demonstrated an essential role of T cells in hair regeneration. The communication network of immune and cutaneous cells around the scaffold was complex, and overall, wound healing occurred as a result of the combined effect of several influencing factors. These findings are potentially applicable in the design and selection of biomaterials for clinical use in wound repair, thereby improving patient outcomes. Limitations of this study include the insufficient disclosure of how T cells regulated hair follicle neogenesis. Meanwhile, the mechanical and biological influences that scaffold structure had on wound closure rate were not fully disclosed. The cellular cross-talk between immune cells and skin cells can be further explored.

## MATERIALS AND METHODS

### Electrospinning of polymer scaffolds

We used PLGA [lactide/glycolide = 75:25, *M*_w_ (molecular weight) = 105 kDa; dispersity, 1.897] produced by Jinan Daigang Biomaterial Co. Ltd. (Shandong, China) and FC (from fish scale and skin) obtained from Sangon Biotech Co. Ltd. (Shanghai, China) to fabricate scaffolds by electrospinning. PLGA [20% (w/v)] and FC [2% (w/v)] solutions, dissolved in 1,1,1,3,3,3-hexafluoro-2-propanol solvent (Aladdin Co. Ltd., Shanghai, China), were loaded into a plastic syringe fitted with a flat-tipped 21-gauge (G) needle (inner diameter, 0.5 mm). A high voltage of 7 kV and a distance of 16 cm were used between the needle and the collector. For the random group, the electrostatically charged fiber was ejected toward the grounded flat collector in the high electric field. For the latticed group, an electroconductive chess-like wire net was used as the collector. For the aligned group, the rotational speed of the collecting drum was set at 2800 rpm. The membranes were immersed in 50 mM 1-ethyl-3-(3-dimehylaminopropyl) carbodiimide hydrochloride/*N*-hydroxysuccinimide and 10 mM MES ethanol solution for 24 hours at 4°C to cross-link FC. Then, membranes were washed three times with 75% ethanol and dried in a vacuum oven for 24 hours. Subsequently, the prepared membranes were sterilized using γ-irradiation for in vitro and in vivo experiments. For PVDF-FC scaffolds, PVDF [30% (w/v)] (FL2005, Flurine, Zhejiang, China) and FC [2% (w/v)] were dissolved in *N*, *N*′-dimethylformamide (Keshi, Chengdu, China). The solvent was loaded into a plastic syringe fitted with a flat-tipped 21G needle. A high voltage of 12 kV and a distance of 13 cm were used between the needle and the collector.

### Characterization of scaffolds

Scanning electron microscopy (SEM; JEOL, JSM-6510LV, Japan) was used to observe the surface morphology of the electrospun membranes. Image-Pro Plus was used to quantitatively measure the fiber diameter and distribution from the obtained SEM images. The surface-wetting behavior of the membranes was characterized by measuring the water contact angles (Chengde Dingsheng, JY-82B, China). Five samples were tested for each type of membrane to obtain an average value. The tensile properties of the membranes were tested under a constant upper clamp at a speed of 15 mm/min. All tensile tests conducted followed the criteria of “Plastics-Determination of tensile properties of films” (GB/T 1040.3-2006, corresponding with ISO 1184-1983).

### Cell culture and cell viability test

L929 mouse fibroblast cells and human oral keratinocytes (HOKs) were used for viability tests. Cells were cultured in a medium containing RPMI 1640 medium (HyClone, Logan, UT, USA) supplemented with 10% fetal bovine serum (Gibco 10270, Brazil), and incubated at 37°C in humidified 5% CO_2_/95% air. Cell viability was determined using the Cell Counting Kit-8 (CCK-8, Dojindo Laboratories, Kumamoto, Japan). Electrospun membranes were cut into squares (edge length, 5 mm) and placed on the bottom of 96-well plates (*n* = 3 for each group). L929 cells and HOK cells were seeded onto membranes at 4 × 10^4^ cells/ml and cocultured with the membranes for 1, 3, and 5 days. Blank wells seeded with an equal number of cells were used as Ctrl. CCK-8 solution (10 μl) was added to each well, and the plates were incubated at 37°C for 1 hour. After incubation, the absorbance at 450 nm was measured to determine cell viability, using a microplate reader (Multiskan, Thermo Fisher Scientific, USA).

### Experimental model

#### Excisional wound model

The protocol of the present experiment was approved by the Institutional Review Board of West China Hospital of Stomatology (no. WCCSIRB-D-2016-022). Animals included Sprague-Dawley male rat at ages from 7 to 8 weeks, C57BL/6 male mice at ages from 7 to 9 weeks (Chengdu Dossy Experimental Animals Co. LTD.), and immunodeficient C57Bl/6 (B6.129-Rag2tm1) mice (Shanghai Model Organisms Center Inc., Shanghai, China). Hair at the surgical area was removed. Full-thickness circular excisional wound (diameter, 6 mm) was created at the dorsal skin of rats/mice. Random, aligned, and latticed electrospun scaffolds were trimmed into circular shape (diameter, 8 mm) and were placed below the wound. The Ctrl group did not receive any implants. A sterile Tegaderm film (3M) was placed above the wound to protect the wound area. Then, annular silicone splints (inner diameter, 8 mm; outer diameter, 12 mm; and thickness, 1 mm) were sutured with the Tegaderm film and underlying skin to minimize the contraction of the dorsal muscle. After healing for 1, 2, and 4 weeks, animals were euthanized for sample harvest. Using the residual wound as center, a round skin sample (diameter, 10 mm) containing all the layers of skin was harvested.

***Model for subcutaneous implant placement***

The surgical area on dorsal skin was shaved and aseptically prepared. Three horizontal incisions of approximately 10 mm were made, and subcutaneous pockets were created for membrane implantation. Then, random, aligned, and latticed scaffolds were placed into the pockets. After implantation, the incisions were sutured with interrupted sutures. After recovering for 3, 7, and 14 days, samples including scaffolds and the whole layer of skin at surgical sites were together harvested.

### Specimen harvest for scRNA-seq

We obtained skin samples by cutting off skin at the wound area (circular, diameter, 10 mm). Subcutaneous tissues were removed, and a total of four samples were harvested in each group. The tissues were digested using the Epidermis Dissociation Kit (Epidermis Dissociation Kit, mouse; Miltenyi Biotec, Bergisch Gladbach, Germany). A gentleMACS Dissociator (Miltenyi) was used to dissociate the epidermis (Program B). The sample was then passed through a 70-μm cell strainer (Corning, Durham, NC, USA), centrifuged at 300*g* for 10 min at 4°C, and resuspended with phosphate-buffered saline (PBS) containing 0.5% bovine serum albumin (BSA). Cells were gently washed twice and stored in an icebox. The dermis samples were first cut into pieces (diameter, <1 mm), mixed with 10 ml of enzyme mix containing type I collagenase (3125 μg/ml; Gibco, Grand Island, NY, USA) and 2.5 ml of trypsin (Gibco, Canada), and then poured into a gentleMACS C Tube. After dissociating the tissue for 37 s (skin mode) using the gentleMACS Dissociator, another 10 ml of the enzyme mix was added. The sample was digested for 2.5 hours at 37°C in a hybridization oven (Peqlab PerfectBlot, Germany). Then, the dermis sample was passed through a 70-μm cell strainer (Corning), centrifuged at 300*g* for 5 min at room temperature, and resuspended in 3 ml of red blood cell lysis buffer (Solarbio, Beijing, China). After 3 min, the cell suspension was centrifuged and gently resuspended in RPMI 1640 medium. Cells were gently washed twice with PBS containing 0.5% BSA and stored in an icebox. The epidermis and dermis cell solutions were mixed, centrifuged, and resuspended with 100 μl of Dead Cell Removal MicroBeads (Miltenyi). After incubation for 15 min at room temperature, the cell suspension was diluted in 3 ml of 1× binding buffer (Miltenyi). LS columns (Miltenyi) and a magnetic stand (Miltenyi) were used to remove dead cells and debris. Negatively selected live cells passed through the column and were resuspended in PBS containing 0.05% BSA. Last, the 10x Genomics Single-Cell Protocol was carried out.

### Single-cell encapsulation and library generation

Single cells were encapsulated in water-in-oil emulsion along with gel beads coated with unique molecular barcodes using the 10x Genomics Chromium Single-Cell Platform. For single-cell RNA library generation, the manufacturers’ protocol was performed. (10x Single Cell 3′ v3) Sequencing was performed using an Illumina 1.9 mode with 94574 reads per cell. We used the Seurat alignment method canonical correlation analysis for integrated analysis (Seurat 3.1.0.).

### RNA-seq analysis

RNA was extracted from tissues using standard methods to make sure samples were strictly controlled for quality. Subsequently, the obtained mRNA was randomly interrupted by divalent cations in New England Biolabs, USA (NEB) fragmentation buffer, and the database was constructed according to the NEB general database construction method or chain-specific database construction method. Upon completion of library construction, a Qubit 2.0 Fluorometer was used for initial quantification, and the library was diluted to 1.5 ng/μl. Then, the insert size of the library was detected using an Agilent 2100 bioanalyzer. The effective concentration of the library (>2 nM) was accurately quantified using real time qPCR to ensure library quality. Last, Illumina sequencing of the libraries was performed. Through z-transformation of fragments per kilobase of transcript per million mapped reads of the selected gene, gene expression was analyzed. The sample size for conventional bulk RNA-seq libraries was fixed at three biological replicates. KEGG (Kanehisa M, 2008) is a database resource for large-scale molecular datasets generated by genome sequencing and other high-throughput experimental technologies (https://www.genome.jp/kegg/pathway.html). We used the cluster Profiler R package to test the statistical enrichment of marker genes in KEGG pathways.

Receptor-ligand analysis was performed via CellChat, a tool that is able to quantitatively infer and analyze intercellular communication networks from scRNA-seq data. CellChat predicts major signaling inputs and outputs for cells and how those cells and signals coordinate for functions using network analysis and pattern recognition approaches. Through manifold learning and quantitative contrasts, CellChat classifies signaling pathways and delineates conserved and context-specific pathways across different datasets.

### Real time qPCR

The harvested samples were cut into pieces and homogenized in TRIzol reagent (Ambion, Carlsbad, CA, USA). The total RNA concentration and ratio were detected using a NanoPhotometer (NP80; Implen, Westlake Village, CA, USA) at wavelengths of 260 and 280 nm. The complementary DNAs were synthesized using the PrimeScript RT reagent kit with gDNA Eraser (Perfect Real Time; catalog no. RR047A, Nojihigashi, Kusatsu, Shiga, Japan) and then amplified using qPCR with specific primers. PCR was performed using a QuantStudio 3 Real-Time PCR System (Thermo Fisher Scientific, Waltham, MA). Each 20 μl of PCR mixture contained 10 μl of TB Green Premix Ex Taq (2×; Tli RNaseH Plus, Takara), 0.4 μl of each PCR forward and reverse primers (10 μM), 0.4 μl of ROX reference dye (50×), 2 μl of template, and 6.8 μl of sterile purified water. Samples were incubated for 1 cycle at 95°C for 30 s, 40 cycles at 95°C for 5 s and 60°C for 34 s, and a final cycle at 95°C for 15 s, 60°C for 1 min, and 95°C for 15 s. Results were analyzed using the comparative cycle threshold (ΔΔCT) method to calculate gene expression fold changes normalized to the levels of Gapdh and Actb gene transcripts. The experiments were performed three times independently (*n* = 3). mRNA primers were listed in table S2.

### Fluorescence-activated cell-sorting analysis

The surface markers of macrophages and their phenotypes were examined using flow cytometry to evaluate the proportion and polarization of macrophages. The in vivo specimens were first cut into pieces (diameter, <1 mm). The tissue was mixed with 10 ml of the enzyme mix containing type I collagenase (3125 μg/ml; Gibco) and 2.5 ml of trypsin (Gibco) and poured into a gentleMACS C Tube. After dissociating the tissue on gentleMACS Dissociator for 37 s (skin mode), another 10 ml of the enzyme mix was added. The sample was digested for 2.5 to 3 hours at 37°C in a rotary machine (Peqlab), passed through a 70-μm cell strainer (Corning), and centrifuged at 300*g* for 5 min at room temperature. Cells were gently washed twice with PBS containing 0.05% BSA and stored in an icebox. Then, the cell solutions were coincubated with antibodies against inducible nitric oxide synthase (iNOS) [phycoerythrin (PE), NBP2-22119PE, Novus], CD68 (Allophycocyanin, ab221251, Abcam), Arg1 (PE-Cyanine7, catalog no. 25-3697-82, Thermo Fisher Scientific), Ly6c (Alexa Fluor 700, catalog no. 128023, BioLegend), CD3 (fluorescein isothiocyanate, catalog no. 100203, BioLegend), and Mrc1 (Alexa Fluor 488, catalog no. 141709, BioLegend) at 1:400 dilution in the dark for 30 min to 1 hour at 4°C (100 μl per antibody per sample). Before the staining of CD3, cells were preincubated with purified anti-CD16/CD32 antibody (catalog no. 101301, BioLegend) (1.0 μg per 10^6^ cells in 100-μl volume) for 5 to 10 min on ice to block Fc receptors. All samples were centrifuged at 450*g* for 5 min at 4°C. Supernatants were removed by aspiration, and the pellets were washed twice with 1 ml of PBS solution containing 0.05% BSA. Fluorescence-activated cell-sorting (FACS) analysis was performed using a NovoCyte flow cytometer (ACEA Biosciences, San Diego, California) and FlowJo 10.5.0. The experiments were performed three times independently (*n* = 3).

### Histological and immunofluorescent staining

The sections were pretreated with 1% BSA in PBS containing 0.1% Triton X-100 for 1 hour, incubated in 1% Tween 20 for 20 min, and washed again with PBS. The sections were subsequently analyzed for Krt10 and Krt5, according to the manufacturers’ instructions. Sections were briefly incubated for 30 min in the dark, and excessive dye was rinsed off using PBS. Sections were then incubated with the antibody isotypes to exclude false-positive staining. Double immunofluorescence staining with primary antibodies against cytokeratin 10 (ab76318, Abcam, 1:150) and cytokeratin 5 (ab52635, Abcam, 1:200) and with secondary antibodies (GB25303 and GB21303, 1:400; Servicebio, Wuhan, China) was performed. The immunostained specimens were further subjected to Hoechst 33258 staining (G1011, Servicebio). At least three parallel sections were observed using ortho-fluorescent microscopy and imaging system (Nikon, Tokyo, Japan). Fluorescence area measurements were conducted at five random sites of regenerated epithelia using CaseViewer 2.1 and Image-Pro Plus 7.0 (*n* = 5).

### Statistical analysis

The statistical significance of in vivo and in vitro histological data, qPCR, and FACS data were analyzed using analysis of variance (ANOVA) at the 95% confidence level using GraphPad Prism 8.0 (GraphPad Software, San Diego, CA, USA), and *P* < 0.05 was considered statistically significant. *P* > 0.05 was marked as NS (not significant). All *P* values less than 0.01 are summarized with two asterisks, *P* values less than 0.001 are summarized with three asterisks, and *P* values less than 0.0001 are summarized with four asterisks. The Pearson’s correlation analysis was conducted in SPSS 16.0 (SPSS Inc., Chicago, IL, USA).

## SUPPLEMENTARY MATERIALS

Supplementary material for this article is available at http://advances.sciencemag.org/cgi/content/full/7/22/eabf0787/DC1
